# Genetic Diversity and Evolutionary Dynamics of Feline Panleukopenia Virus in China: Phylogenetic Analysis and Substitution Patterns in NS1 and VP2 Proteins

**DOI:** 10.3390/v18050562

**Published:** 2026-05-15

**Authors:** Zihan Ye, Danni Wu, Xueru Jiang, Lina Liu, Guoliang Luo, Zhenjun Wang, Yuening Cheng, Erkai Feng

**Affiliations:** 1 Key Laboratory of Economic Animal Diseases, Ministry of Agriculture, Institute of Special Animal and Plant Science, Chinese Academy of Agriculture Science, Changchun 130112, China; youzi0915ye@163.com (Z.Y.); a13849938492@163.com (X.J.); liulina@caas.cn (L.L.); luoguoliang@caas.cn (G.L.); wangzhenjun@caas.cn (Z.W.); 2 Department of Preventive Veterinary Medicine, College of Animal Medicine, Jilin Agricultural University, Jilin 130118, China; 13321435868@163.com

**Keywords:** Feline panleukopenia virus, genetic divergence, synonymous substitution, non-synonymous substitution

## Abstract

Feline panleukopenia virus (FPLV) is the primary causative agent of a highly contagious and often fatal disease affecting domestic cats and other felids. The increasing isolation of species-specific FPLV variants from multiple host species has garnered considerable attention, highlighting the need to investigate their genetic diversity. In this study, three FPLV isolates were obtained and phylogenetically classified into two distinct FPLV-China groups within separate clusters. Compared to the prototype FPLV (M38246.1), these isolates exhibited seven amino acid substitutions in the NS1 (*n* = 6) and VP2 (*n* = 1) proteins. Further analysis of 157 NS1 sequences and 947 VP2 sequences retrieved from the NCBI database revealed 113 and 479 synonymous substitutions and 71 and 279 non-synonymous substitutions, respectively. Notably, the majority of these substitutions occurred as single events (57% in NS1, 40/71; 55% in VP2, 153/279) or were present in no more than five FPLV sequences (23% in NS1, 16/71; 32% in VP2, 89/279). However, three non-synonymous substitutions in the NS1 protein (Ile443Val, His595Gln, and Val596Leu) were detected in more than half of the 157 sequences analyzed. In the VP2 protein, six non-synonymous substitutions (Ala91Ser, Thr101Ile, Val232Ile, Lys93Asn, Asp323Asn, and Val562Leu) were each found in 20 to 40 FPLV sequences. Furthermore, ten sites in the NS protein and 224 sites in the VP2 protein exhibited both synonymous and non-synonymous substitutions simultaneously. Additionally, 75 sites in VP2 harbored multiple non-synonymous substitutions. These findings provide valuable insights for future research on the genetic determinants and vaccine development of FPLV.

## 1. Introduction

Feline panleukopenia virus (FPLV), also known as feline parvovirus, is the prototype of a group of antigenically related parvoviruses belonging to the species *Protoparvovirus* carnivoran 1 of the genus *Protoparvovirus* within the family *Parvoviridae* [1]. It primarily affects domestic cats and endangered carnivores, posing a significant threat to the Chinese pet industry and wildlife protection [2,3].

FPLV is a non-enveloped, single-stranded DNA virus with a genome of approximately 5200 base pairs (bp) [4]. This virus encodes four proteins (NS1, NS2, VP1, and VP2) through alternative splicing of two open reading frames (ORFs). NS1 is the key non-structural protein and plays a vital role in regulating virus replication and assembly, suppressing host innate immunity, and determining viral pathogenicity [5]. Meanwhile, the structural protein VP2, which constitutes 90% of the viral capsid, exerts a pivotal influence on the host range selection, immunogenicity, and evasion of the host immune response [6,7].

The evolutionary pattern of FPLV was once considered relatively conserved, with just a single serotype [8,9], unlike canine parvovirus type 2 (CPV-2, CPV-2a/2b/2c, and new CPV-2a/2b/2c). However, in recent years, numerous species-specific FPLV variants with non-synonymous substitutions in the VP2 [1,4,10,11] or NS1 protein [6] have been successively isolated from multiple other host species, including monkeys [12], giant pandas [13,14], dogs [11], minks [15], and tigers [3,16,17,18,19]. While cross-species transmission of FPLV has been documented [5,20], and the specific genetic determinants enabling host adaptation—particularly the functional roles of non-synonymous substitutions in NS1 and VP2 proteins—remain poorly characterized. Non-synonymous substitutions in these proteins are therefore prime candidates for driving host adaptation, yet their distribution patterns and evolutionary dynamics across global FPLV populations have not been systematically studied. All these have attracted significant attention due to the emergence of these species-specific FPLV variants.

Therefore, in this study, we conducted a comparative analysis of all NS sequences and VP2 sequences of FPLVs deposited in the NCBI database (up to date 2 April 2025) to gain a comprehensive understanding of genetic variation among different FPLV strains and then provide valuable insights for genetic research and vaccine development of FPLV.

## 2. Materials and Methods

### 2.1. Samples Collection

Between December 2017 and May 2020, a total of 16 clinical samples (fecal specimens or rectal swabs) were collected from cats suspected of being infected with FPLV in Shijiazhuang (*n* = 6) and Changchun (*n* = 10), the capital cities of Hebei and Jilin provinces in China. These cats exhibited clinical signs including diarrhea, vomiting, or dehydration. Information regarding age, breed, and vaccination status was not recorded. The fecal samples were resuspended in 0.5 mL of sterile phosphate-buffered saline (PBS, 1×) and stored at −20 °C.

Fecal specimens or rectal swabs were homogenized in serum-free Minimum Essential Medium (MEM) (*w*/*v*, 1:9) and subsequently clarified by centrifugation at 10,000× *g* for 30 min using a Rotator type F-34-6-38 centrifuge (Eppendorf, Hamburg, Germany). The supernatant was treated with penicillin and streptomycin at 4 °C overnight until further use.

### 2.2. PCR Identification

Viral DNA was extracted from 200 µL of fecal supernatants using the Takara Mini BEST Viral RNA/DNA Extraction Kit Ver. 5.0 (Takara Bio Inc, Beijing, China) according to the manufacturer’s instructions. PCR amplification targeting the full-length VP2 gene was performed using a primer set (VP2-F:5′-CTTACGCTGCTTATCTTCGCTCTGG-3′; VP2-R:5′-TTTTGGTCCTTAACATATTCTAAGGGCAA-3′ [4,21] and a Prime STAR^®^ Max DNA polymerase (Takara Bio Inc, Beijing, China). The standardized PCR systems (25 µL) contained 1.5 µL of viral DNA, 12.5 µL of a Prime STAR^®^ Max DNA Premix (2×), 1.5 µL each of the primers, and 8 µL of sterile distilled water. The PCR was performed with the following steps: initial denaturation at 98 °C for 120 s, followed by 35 cycles of denaturation at 98 °C for 15 s, annealing at 58 °C for 15 s, and extension at 72 °C for 150 s, with a final extension at 72 °C for 600 s. The PCR products were analyzed by electrophoresis using a 1% agarose gel and then sequenced by Comate Bioscience Co., Ltd. (Changchun, Jilin, China).

### 2.3. Virus Isolation

Feline kidney cells monolayers (F81) (CVCL_9259), obtained from the American Type Culture Collection (ATCC, Gaithersburg, MD, USA), were inoculated with the treated supernatant (1:10 ratio) and incubated in a 5% CO_2_ incubator at 37 °C for 1 h, shaking every 20 min. After incubation, the samples were washed three times with PBS (pH 7.4), and 2% fetal bovine serum MEM was added. The cells were then incubated at 37 °C in incubators with 5% CO_2_, with the virus being passaged up to times in the cells until cytopathic effects (CPEs) were observed. The cultures were monitored daily for CPE, such as crumping and detachment. Once CPE reached 80–90%, cell cultures were frozen at −80 °C, thawed three times, and centrifuged at 4 °C and 13,000× *g* for 10 min, and the supernatant was collected as viral stock and stored at −80 °C for future use.

### 2.4. Direct Immunofluorescence Assay

A direct immunofluorescence assay (DIFA) was carried out to confirm the presence of FPLV in the samples. Briefly, F81 cells were infected with the virus and incubated for 48 h. After that, the cells were fixed with precooled acetone for 45 min at 4 °C. Next, a 1 h blocking step was performed using 1% bovine serum albumin (BSA) at 37 °C. Subsequently, the cells were incubated with an FITC-conjugated monoclonal antibody (Mab) specific for FPLV (VMRD, Inc., Pullman, WA, USA) for 1 h at 37 °C. Ultimately, the cells were examined under a fluorescence microscope (Leica AF6000, Wetzlar, Germany).

### 2.5. Amplification of the Full-Length Genome Sequences of Three FPLVs

DNA was extracted from three FPLV isolates using the Viral DNA Extraction Kit (Takara, Beijing, China), following the manufacturer’s instructions. The full-length genomes of three FPLVs were amplified using specific primers described by Chen et al. [22]. The primer sequences are detailed in Table 1.

The U and Y structural regions of the viral DNA were amplified using primers (U1-F/R, U2-F/R, Y1-F/R, and Y2-F/R) and Prime STAR HS DNA polymerase (Takara, Beijing, China). Briefly, 2 μL of viral DNA was mixed with 12.5 μL 2× Prime STAR GC buffer, 2 μL of dNTPs (2.5 μM each), 1.5 μL of forward primers (Y1-F, Y2-F, U1-F, and U2-F, 10 pmol/μL each), and 5 μL of ddH_2_O. The mixture was boiled for 5 min and then immediately chilled on ice for 10 min [22]. Subsequently, 0.5 μL of PrimeSTAR^®^ HS DNA Polymerase was added, and the mixture was incubated at 72 °C for an additional 3 min. Finally, 1.5 μL of reverse primers (Y1-R, Y2-R, U1-R, and U2-F) were added. All reactions were performed in a total volume of 25 µL.

The inverted terminal repeat (ITR) regions were amplified under the following conditions: initial denaturation at 98 °C for 5 min, followed by 40 cycles of denaturation at 98 °C for 10 s, annealing at 60 °C for 10 s, and extension at 72 °C for 10 s, with a final extension at 72 °C for 10 min. The PCR products were further sequenced by Comate Bioscience Co., Ltd. (Changchun, Jilin, China).

The middle regions of the FPLV genome were amplified using specific primers (M1-F/R, M2-F/R, and M3-F/R) and a PrimeSTAR^®^ Max DNA Polymerase (Takara, Beijing, China) under the following conditions: initial denaturation at 98 °C for 5 min; followed by 40 cycles of denaturation at 98 °C 10 s, annealing at 55 °C for 10 s, and extension at 68 °C for 20 s; with a final extension at 68 °C for 10 min. The PCR fragments were separated by agarose gel electrophoresis and purified using the DNA Gel Extraction Kit (Axygen, Hangzhou, China).

### 2.6. Sequencing and Phylogenetic Analysis

All PCR products were subsequently sequenced by Comate Bioscience Co., Ltd. (Changchun, Jilin, China). The sequences of all fragments were assembled using SeqMan software (version 11.0; DNASTAR Inc., Madison, WI, USA). The nearly full-length sequences obtained were submitted to the National Center for Biotechnology Information (NCBI) and then subjected to BLASTn analysis to determine the homology percentage between our isolates and all other FPLV isolates with full-length genomes or nearly full-length genomes in the GenBank database.

The 104 parvovirus genomes exhibiting the highest homology to our three Chinese FPLV isolates were retrieved from the NCBI database. Together with our three Chinese FPLV isolates, these sequences (Table S1) were used to construct a phylogenetic tree using MEGA software (version 11.0, Mega Limited, Auckland, New Zealand) to compare the phylogenetic relationships among our isolates and the 100 most closely related reference full-length parvovirus genome sequences. The maximum likelihood (ML) method was employed with 1000 bootstrap replicates.

### 2.7. Mutations Analysis of NS1 and VP2 Sequence of FPLV Isolates

Based on the search terms “Feline panleukopenia virus[title] AND (NS1[title]” or “Feline parvovirus[title] AND (NS1[title]” with the filter “sequence length = 2007 bp”, and, similarly, using the search terms “Feline panleukopenia virus[title] AND (VP2[title] or capsid[title])” or “Feline parvovirus[title] AND (VP2[title] or capsid[title])” with the filter “sequence length = 1755 bp” to exclude partial sequences, a total of 157 reference complete NS1 sequences and 947 references complete VP2 sequences, along with metadata of FPLV isolates such as host, date and country of collection (including those of three FPLV isolates), were retrieved from the GenBank database (accessed on 2 April 2025; see Tables S2 and S3). Mutations analysis was performed based on the NS1 and VP2 nucleotide sequences (2007 and 1755 base pairs, respectively) and deduced amino acid sequences (669 and 585 amino acids, respectively). Sequence alignments and pairwise sequence comparisons were carried out using the MEGA software (version 11.0, Mega Limited, Auckland, New Zealand) and Microsoft Excel.

## 3. Results

### 3.1. PCR Identification of Clinical Samples

**Figure 1:**
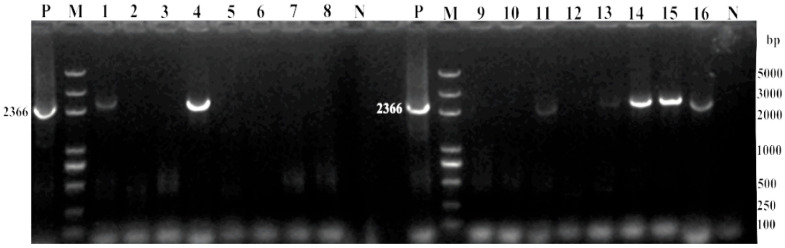
PCR identification of clinical samples. M: DL5000 DNA marker; P: positive control; N: negative control; 1–16: clinical samples 1–16.

A total of 7 out of 16 (44%) clinical samples tested positive for FPLV by a conventional PCR assay targeting the VP2 gene, indicating FPLV infection in cats. Among them, three clinical samples yield good quality sequence and showed high-intensity, clear, and bright bands at 2366 bp (Figure 1).

### 3.2. Isolation and Identification of FPLV

**Figure 2:**
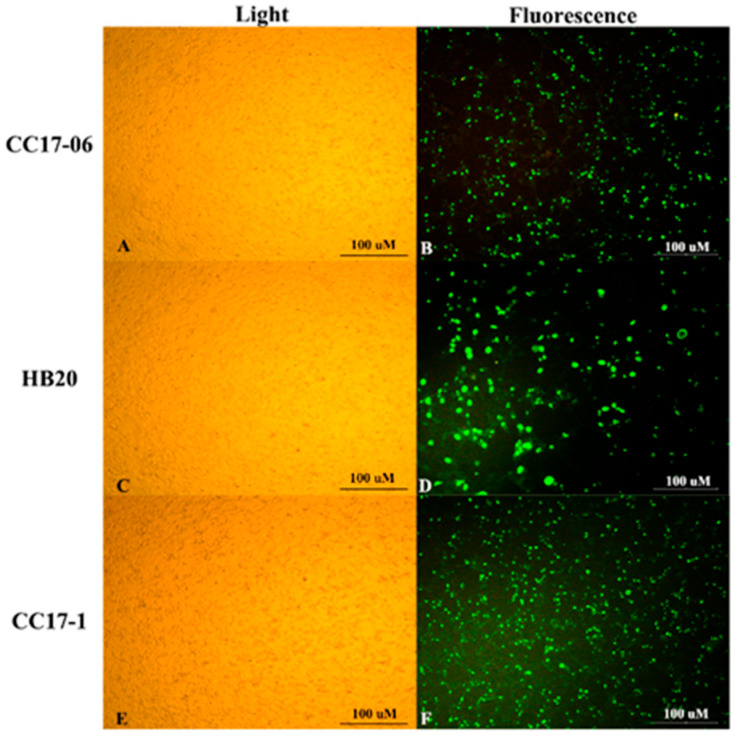
Cytopathic effect and direct immunofluorescence assay of three FPLV strains on F81cells. (**A**) F81 cell infected with FPLV-CC706 (light); (**B**) F81 cell infected with FPLV-CC706 (fluorescence); (**C**) F81 cell infected with FPLV-HB20 (light); (**D**) F81 cell infected with FPLV-HB20 (fluorescence); (**E**) F81 cell infected with FPLV-CC17-1 (light); (**F**) F81 cell infected with FPLV-CC17-1 (fluorescence).

Three PCR-positive clinical samples induced cytopathic effects (CPEs) on F81 cells, marked by the disintegration of infected cells (Figure 2A,C,E). Direct immune-fluorescence assay via the Anti-Feline IgG (H&L) FITC Conjugate Affinity Purified (CJF-F-FLG-AP, VMRD, Inc., Pullman, WA, USA) could also detect these effects (Figure 2B,D,F). The three virus isolates were further designated as FPLV-CC1706, FPLV-HB20, and FPLV-CC17-1, respectively.

### 3.3. Construction of Nearly Full-Length Genome Sequences of Three Chinese FPLV Isolates

**Figure 3:**
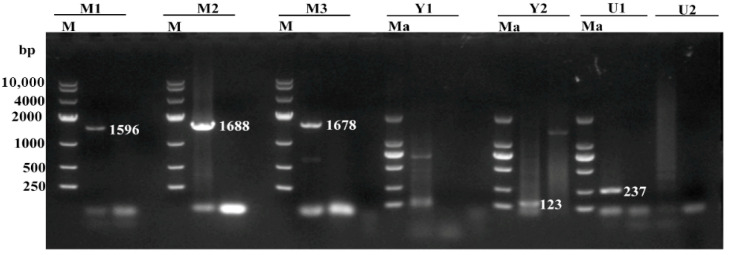
The nearly full-length genome amplification of FPLV strains. M: DL 10,000 DNA Marker; M1: PCR fragment (primer M1); M2: PCR fragment (primer M2); M3: PCR fragment (primer M3); Ma: DL 2 000 DNA Marker; Y1: PCR fragment (primer Y1); Y2: PCR fragment (primer Y2); U1: PCR fragment (primer U1); U2: PCR fragment (primer U2).

The middle regions and part of the 3′ inverted terminal repeats (ITRs) (Y2) and 5′ ITRs (U1) of FPLV clinical strains were successfully amplified in this study (Figure 3). The amplified PCR products were 1596 bp (M1), 1688 bp (M2), 1678 bp (M3), 23 bp (Y2), and 237 bp (U1), respectively, and their sizes were consistent with expectations. The resulting sequences were assembled and deposited in GenBank and were assigned the accession numbers ON630416.1 (FPLV-CC1706), ON630417.1 (FPLV-HB20), and ON630418.1 (FPLV-CC17-1), respectively. However, it is a pity that we did not succeed in amplifying the gene fragments of Y1 and U2 in this study, which both belong to the end of the 3′ or 5′ ITRs region in the FPLV genome.

### 3.4. Phylogenetic Analysis of Nearly Full-Length Genome of Three Chinese FPLV Isolates

Blast searches were performed on the nearly full-length genome sequences of three Chinese FPLV isolates against the NCBI database. A total of 100 parvovirus strains identified through BLAST searches (https://blast.ncbi.nlm.nih.gov/Blast.cgi, accessed on 1 May 2026) were selected, which exhibited 98.2–99.92% nucleotide identity to the three nearly full-length genome sequences: ON630416.1, 98.33–99.76%; ON630417.1, 98.22–99.75%; and ON630418.1, 98.23–99.92%.

**Figure 4:**
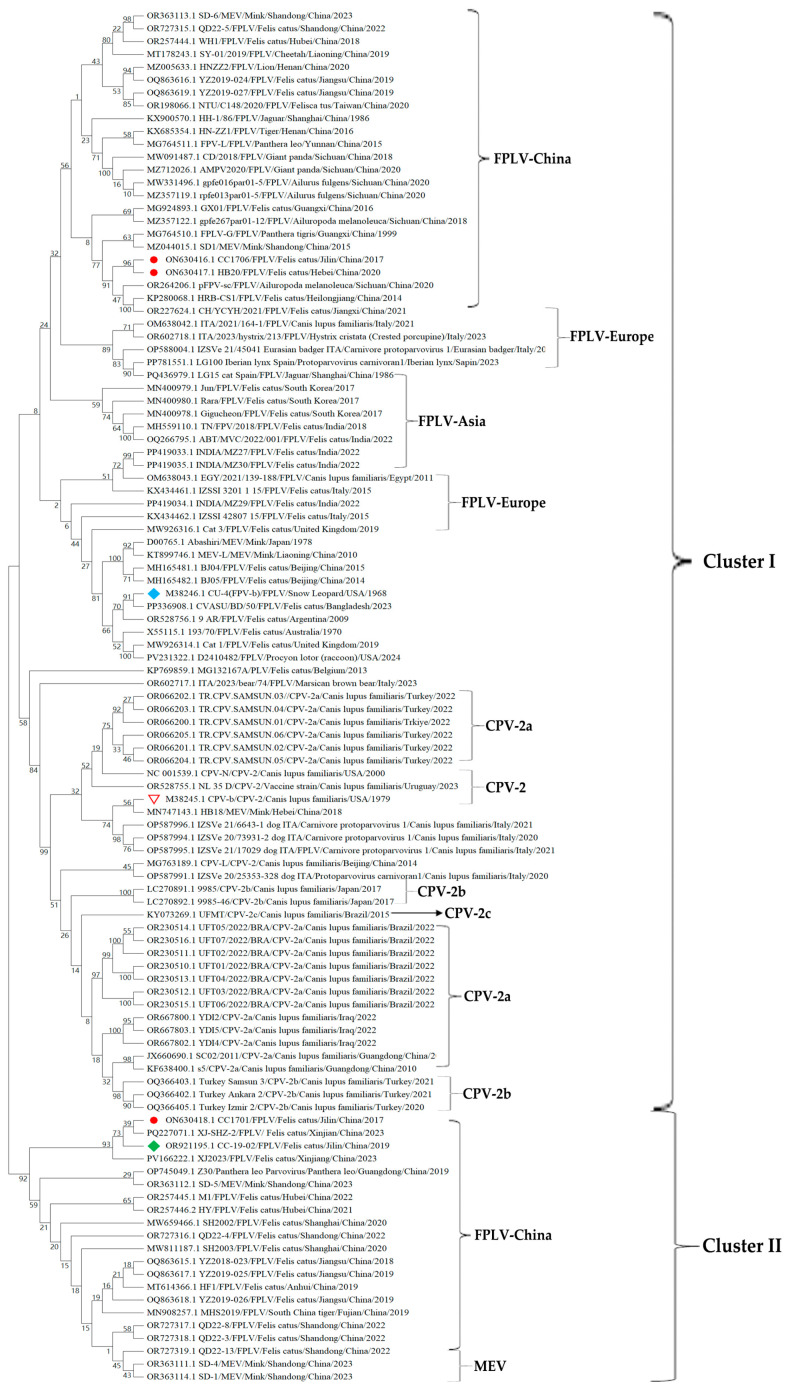
Phylogenetic analysis between the three Chinese FPLV strains and other known parvoviruses with complete genomes.

A phylogenetic tree was constructed based on the 3 FPLV strains and 104 most related parvoviruses, including 8 mink enteritis viruses (MEVs), 18 CPV-2a, 5 CPV-2b, 1 CPV-2c, 10 CPV-2, and 65 FPLVs (Figure 4). The phylogenetic analysis indicated that all parvoviruses were grouped into two large clusters (cluster I and II), which exhibited strong characteristics of virus types and geographical regions (Figure 4). Two our FPLV isolates (ON630416.1 and ON630417.1), located in cluster I, formed a small branch that subsequently clustered with numerous Chinese FPLV isolates, some of which were derived from rare and protected animals, like *Ailuropoda melanoleuca* (giant panda) (OR264206.1, MZ357122.1, MZ712026.1, and MW091487.1), *Ailurus fulgens* (red panda) (MZ357119.1 and MW331496.1), *Panthera tigris* (tiger)/jaguar (MG764510.1, KX685354.1, and KX900570.1), and *Panthera leo* (lion) (MG764511.1 and MZ005633.1).

The remaining FPLV isolates (ON630418.1) were located in cluster II and formed an independent branch with two FPLV strains isolated from northeastern (OR92195.1) or northwestern (PQ227071.1) China (Figure 4). This isolate also formed a larger cluster with numerous Chinese FPLV isolates derived primarily from the original host, *Felis catus* (Figure 4). All three FPLV isolates in this study showed significant genetic distance from canine parvovirus (CPV-2a, CPV-2b, and CPV-2c), as well as from foreign FPLV isolates from America, Europe, or other Asian countries.

### 3.5. Molecular Analysis of NS1 Gene and VP2 Gene of Three Isolated FPLVs

**Figure 5:**
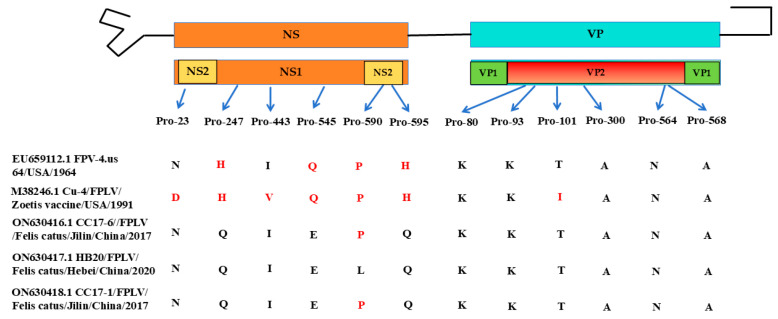
Comparative genome analysis of 3 FPLV isolates and the prototype FPLV strain. Red letters indicated the specific amino acids at specific positions in the NS and VP2 protein of the protype virus strain of FPLV.

After conducting a comparative genome analysis with the FPLV prototype strain (M38246.1) and the first isolated FPLV based on current knowledge, six non-synonymous substitutions (Asp23Asn, His247Gln, Val443Ile, Gln545Glu, Pro590Leu, and His595Gln) were identified in the NS1 gene, and one non-synonymous substitution (Ile101Thr) was found in the VP2 gene of the three FPLV isolates (Figure 5).

### 3.6. Genetic Variance Analysis of NS1 Gene of FPLV Isolates

**Figure 6:**
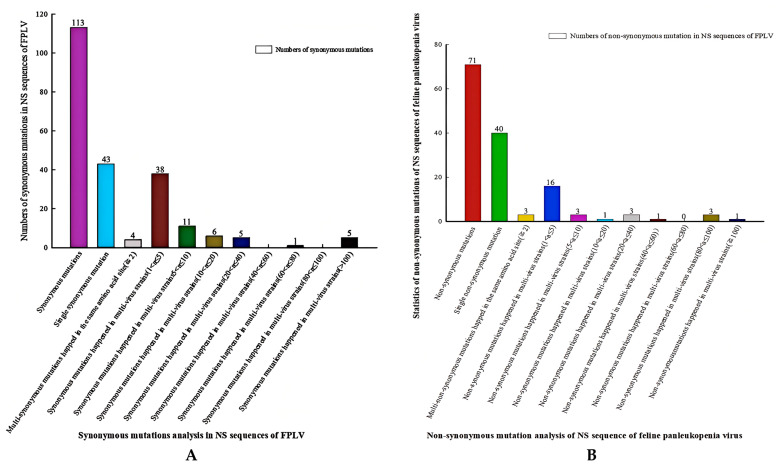
Statistical analysis of non-synonymous and synonymous substitutions of NS1 sequences of FPLVs.

A total of 157 complete NS1 sequences (2007 bp) of FPLVs deposited in the NCBI database (up to April 2025) were analyzed for genetic variation at the nucleotide and amino acid level using MEGA software. This analysis revealed 174 nucleotide substitution sites, of which 71 exhibited non-synonymous substitutions, 113 exhibited synonymous substitutions, and 10 sites (60, 110, 207, 249, 417, 503, 517, 554, 584, and 586) underwent both types of substitutions concurrently (Figure 6; Tables S4 and S5).

Among the 113 synonymous substitutions, 43 were single-event substitutions (occurring in only one virus strain), while 4 amino acid (aa) sites (17, 103, 214, and 479) experienced two or more synonymous substitutions. Further analysis revealed that 38 synonymous substitutions occurred in no more than 5 FPLVs, 11 in at most 10 FPLVs, 6 in fewer than 20 FPLVs, 5 in fewer than 40 FPLVs, and 5 synonymous substitutions (522 nt, A→G; 1467 nt, C→T;1686 nt, C→T; 1926 nt, A→G, and 1959 nt, T→C) occurred in more than 100 FPLVs (Figure 6A; Table S4).

**Figure 7:**
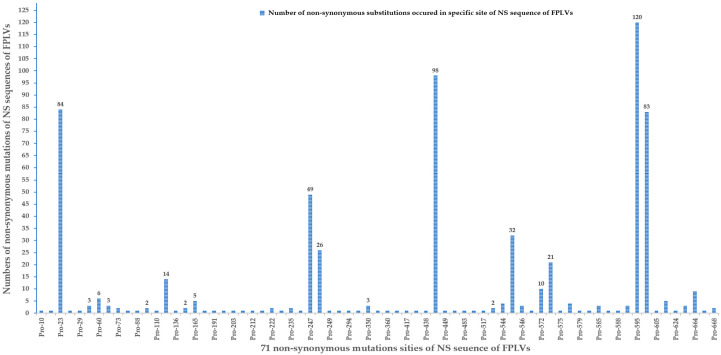
Statistical analysis of 71 non-synonymous substitutions of NS1 sequences of FPLVs.

In total, 71 non-synonymous substitutions were identified in 157 NS1 sequences of FPLVs (Figure 6B). The number of FPLV isolates experiencing each of the 71 non-synonymous substitutions is depicted in Figure 7. Single-event substitutions (occurring in only one FPLV strain) accounted for a relatively large proportion, reaching 57.7% (40/71) (Figure 6B and Figure 7). A total of 16 non-synonymous substitutions occurred in no more than 5 FPLVs, and 3 non-synonymous substitutions (Thr60Ala, Glu572Lys, and Arg664Gln) occurred in no more than 10 FPLVs. Additionally, 3 non-synonymous substitutions (Ile443Val, His595Gln, and Val596Leu) were detected in more than 80 FPLV strains. Notably, 3 amino acid (aa) sites (Gln545Glu, Lys or Val; Pro590Arg, or Leu; Asp616Asn, or Gly) experienced 2–3 non-synonymous substitutions, indicating the genetic instability of FPLVs (Table S5).

So far, very few studies have focused on the function of these non-synonymous substitutions; however, recently, a Chinese researcher reported a FPLV variant carrying a non-synonymous substitution at the 588th amino acid site (Ser588Asn) (GenBank No. OR257445.1), which helps the virus escape neutralization and augmenting viral replication [6]. The Lys80 substitution is essential for FPLV replication in feline cells, while Asn93 and Asn323 are essential for CPV replication [23].

### 3.7. Genetic Variance Analysis of VP2 Gene of FPLV Isolates

Following a comprehensive analysis of genetic variation in the VP2 protein among 947 complete VP2 sequences (1755 bp) of FPLVs deposited in the NCBI database (as of 2 April 2025), we identified a total of 479 synonymous substitutions and 279 non-synonymous substitutions.

**Figure 8:**
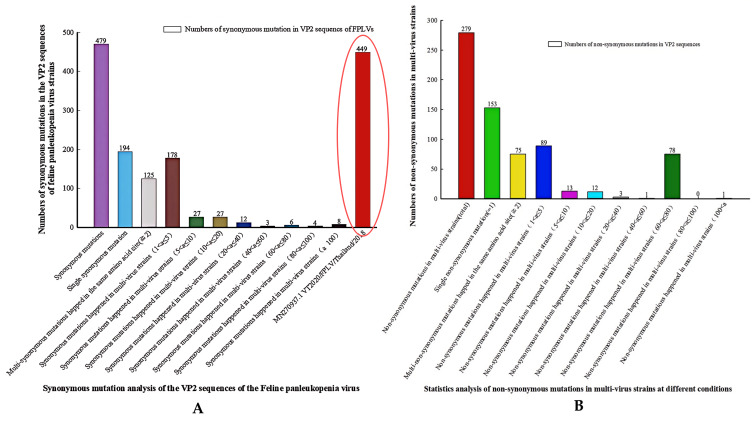
Statistical analysis of non-synonymous and synonymous substitution of VP2 sequences of FPLVs.

Among them, 224 amino acid (aa) sites experienced both non-synonymous and synonymous substitutions concurrently. Seventy-five aa sites (5, 18, 21, 80, 91, 324, among others) harbored multiple non-synonymous substitutions (Tables S6 and S7, Figure 8). Notably, one FPLV isolate obtained from a dog in Thailand in 2018 [24] (GenBank No. MN270937.1) exhibited 93.7% of the 479 synonymous substitutions (449/479) (Figure 8A, red ellipse).

**Figure 9:**
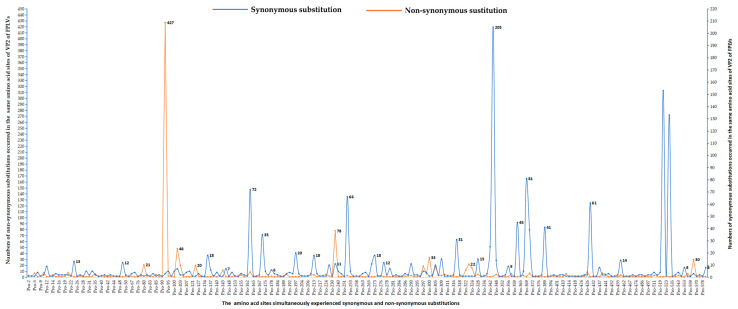
Statistical analysis of 224 amino acid sites simultaneously experienced non-synonymous and synonymous substitution.

**Figure 10:**
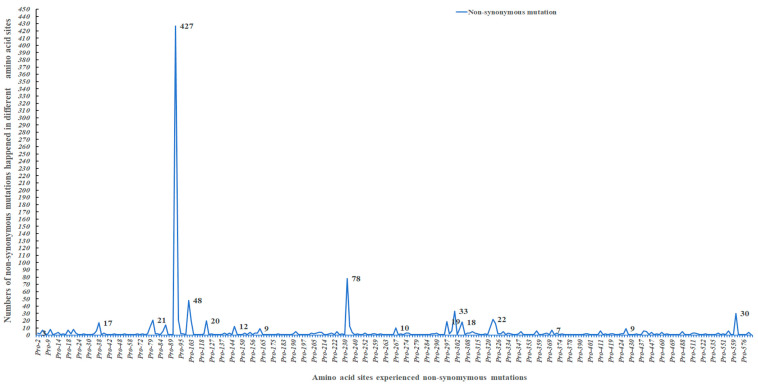
Statistical analysis of 279 non-synonymous substitutions of VP2 sequences of FPLVs.

Further analysis of the 279 non-synonymous substitutions revealed that most were single non-synonymous substitutions (153/279) or occurred in no more than 5 FPLV strains (89/279) (Figure 8B, Figure 9 and Figure 10, Table S7). However, three non-synonymous substitutions (Ala91Ser, Thr101Ile, and Val232Ile) were detected in more than 40 FPLV strains, and three additional substitutions (Lys93Asn, Asp323Asn, and Val562Leu) occurred in 20–40 FPLV strains. Twelve non-synonymous substitutions (Lys80Arg, Met87Leu, Val103Ala, His234Tyr, Ser297Ala, Ala300Pro or Gly, Asp305Tyr, and Tyr324lle) were found in 10–20 FPLV strains (Figure 8B, Figure 9 and Figure 10, Table S7).

## 4. Discussion

In recent years, an increasing number of species-specific FPLVs carrying one or more non-synonymous mutations in either structural protein or non-structural protein have been frequently isolated from multiple host species [1,4,10,11,14,25]. More importantly, some mutations alter the host range [11,14,25,26,27], while others affect viral replication or pathogenicity [6,9,10]. Therefore, it is urgent to obtain a comprehensive understanding of genetic variation among different FPLV strains to provide valuable information for genetic research and vaccine development.

In this study, three domestic FPLV isolates were obtained and classified into two distinct FPLV-China groups distributed across two separate large clusters. Two isolates (ON630416.1 and ON630417.1) showed close evolutionary relationships with FPLV isolates derived from rare and protected animals (Figure 4). Our FPLV isolates showed substantial genetic distance from both canine parvovirus and foreign FPLV isolates from America, Europe, or other Asian countries. These findings highlight the necessity of screening local vaccine strains.

Furthermore, compared with the prototype FPLV (M38246.1) and the first FPLV isolate deposited in the NCBI database (EU659112.1), a total of seven non-synonymous mutations were identified: six in the NS protein (Asp23Asn, His247Gln, Val443Ile, Gln545Glu, Pro590Leu, and His595Gln) and one in the VP2 protein (Ile101Thr) (Figure 5). Unfortunately, the function of these mutations remains unclear. To our knowledge, the only documented report of a non-synonymous mutation in the NS1 protein (Ser588Asn) was published in 2024 [6]; this mutation enhances viral replication ability and expression of interferon-stimulated genes (ISGs). The Ile101Thr mutation was first reported in a dog-derived FPLV by researchers from Taiwan, China [11], and was subsequently identified in FPLVs derived from captive Siberian tigers [16,17,19]. However, following a comprehensive analysis of genetic variation in 947 VP2 sequences, we found that the 101st amino acid in the VP2 protein of the first FPLV isolate (EU659112.1), isolated in 1964, was threonine (Thr), with 101Thr present in 95% (899/947) of all FPLVs. The first 101Ile FPLV, designated TU8 (AB000070.1), was isolated from Japan in 1976 [9], and has been continuously detected in clinical samples from the 1980s to the present. Notably, 101Ile mutations are much more prevalent in canine parvovirus (CPV).

Moreover, a comprehensive analysis of genetic variation in the NS1 and VP2 sequences of FPLVs at the nucleotide and amino acid level was carried out in this study by using the method of mathematical statistics based on all the NS1 sequences (n = 157) and VP2 sequences (n = 947) of FPLVs deposited in the NCBI database. In total, 71 and 279 non-synonymous mutations were identified in the NS1 and VP2 sequences, respectively. Notably, most of these non-synonymous mutations were single events (>50%) or occurred in a small proportion of FPLVs (<30%), whereas some mutations were present in more than half of the sequences analyzed.

Additionally, three non-synonymous mutations (Ile443Val, His595Gln, and Val596Leu) in the NS1 protein were detected in more than half of all NS1 sequences (80/157). At three amino acid sites, 2–3 non-synonymous mutations occurred simultaneously (Gln545Glu, Lys or Val; Pro590Arg, or Leu; Asp616Asn, or Gly). These findings highlight the need for further research on these non-synonymous mutations in the NS1 protein.

Four amino acid mutations (Ala91Ser, Thr101Ile, Val232Ile, and Val562Leu) were detected in more than 40 FPLV strains. These mutations were speculated to play a vital role in virus–receptor interaction, either by changing the surface charge of VP2 protein or altering its conformation [10,11,28]. The Ala91Ser FPLV variant, accounting for 45% of all FPLV isolates analyzed in this study (422/947), was first identified in a tiger-derived FPLV (DQ099431) in China in 2006. It subsequently spread sporadically to Europe [29,30], South Korea [31], and South America [32] during 2006~2008, and re-emerged in China in 2017. The prevalence of Ala91Ser FPLV variants increased from 10% in 2017 to 87% in 2022–2023 [4]. Furthermore, after 2023, this variant spilled over to Southeast and South Asia [33,34]. To date, this FPLV variant continues to be isolated from clinical samples [7,35]. In silico structural analysis predicted that the Ala91Ser mutation in VP2 would extend the disordered region (residues 91–95), compromising the receptor–binding interface and consequently driving the altered host-range tropism of the FPLV variant [4,10,28,36].

Other FPLV variants isolated from different hosts mostly carried typical non-synonymous mutations in VP2 proteins, such as the Gly299Glu mutation in captive giant panda-derived FPLVs [13,14,23] and cat-derived FPLVs [37], the Ala300Pro mutation in dog-derived FPLVs [25], and the Val300Ala mutation in mink-derived FPLVs [15]. The frequency of Gly299Glu and Ala300Pro is relatively low, at 0.3% (3/947) and 1.6% (15/947), respectively. VP2 position 300 is a critical determinant in cross-species transfer of parvovirus of different carnivore hosts, and mutations at this residue alter the virus’s ability to bind the transferrin receptor (TfR) and infect different carnivore hosts [26]. Gly299Glu, located in loop 3 of VP2 protein, alters the structure of the neutralizing B cell epitope, further affecting the antigenicity of FPLV [14]. Earlier work demonstrated that introducing this mutation into a CPV-2 infectious clone severely attenuates viral infection in canine cells [26]. These mutations warrant extensive investigation to elucidate their functions in future studies, which will provide valuable information for genetic studies and vaccine development of FPLV.

## 5. Conclusions

This study systematically characterized the genetic diversity of FPLV by analyzing 1104 publicly available sequences, identified seven novel substitutions in Chinese FPLV isolates, and comprehensively mapped synonymous and non-synonymous mutations across NS1 and VP2 proteins. Notably, three non-synonymous substitutions (Ile443Val, His595Gln, and Val596Leu) in NS1 were highly prevalent, while six substitutions in VP2 (Ala91Ser, Thr101Ile, Val232Ile, Lys93Asn, Asp323Asn, and Val562Leu) were associated with species-specific variants, reflecting their importance in host adaptation and for rational vaccine design. This study is primarily based on computational sequence analysis, and the functional consequences of identified mutations remain to be validated experimentally.

## Figures and Tables

**Table 1:** Primers for amplification of the full-length genome of FPLV.

Primer Name	Sequence (5′-3′)	Position	Length (bp)
Y1-F	ATCATTCTTTAGAACCAACTGACCAAG	1–27	69
Y1-R	GCAGCGCGCGCAGCGCGCGTCAT	47–69	
Y2-F	GCTGCGCGCGCTGCCTAC	56–73	123
Y2-R	AACCACGCCCACAATTAGCCCG	166–187	
M1-F	TTGTGTGTTTAAACTTGGGC	134–153	1596
M1-R	GTTGTCATAATTACTGGAGTTGG	1707–1729	
M2-F	GGAAGTAAGCAAATTGAACC	1689–1709	1688
M2-R	AAACCCAATGTCTCAGATCTC	3356–3376	
M3-F	CTGTTTCAGAATCTGCTACTC	3241–3261	1678
M3-R	GGTTAGTTCACCTTATAGACAG	4897–4918	
U1-F	TAATGTATGTTGTTATGGTGTGG	4792–4814	237
U1-R	CTACGCGGTCTGGTTGATTAAGC	5006–5029	
U2-F	GCGGTCTGGTTGATTAAGC	5027–5046	96
U2-R	AAGTATCAATCTGTCTTTAAGGGG	5100–5123	

## Data Availability

The original contributions presented in this study are included in the article. Further inquiries can be directed to the corresponding author.

## Supplementary Materials

The following supporting information can be downloaded at: https://www.mdpi.com/article/10.3390/v18050562/s1, Table S1: 107 complete genome for phylogenic tree; Table S2: 157 reference complete NS sequences of FPLVs; Table S3: 947 reference complete VP2 sequences of FPLVs; Table S4: Synonymous mutation analysis of NS sequences of FPLVs(157);Table S5: Non-synonymous mutation analysis of NS sequences of FPLVs(157); Table S6:Synonymous mutations analysis of VP2 sequences of FPLVs(947); Table S7: Non-synonymous mutations analysis of VP2 sequences of FPLVs(947).

## Abbreviations

The following abbreviations are used in this manuscript:

FPLV	Feline panleukopenia virus
aa	Amino acid
TfR	Transferrin receptor

## References

[1] Safwat M.S., Xi C., El-Sayed M S., Anwer A.Z., Ali M.E., Abdallah E.S.A., Amer H.M., Saeed O.S., Khalil G.M., Abdelhaleem S.W. (2025). Emergence, Surge, and Fading of the Novel Feline Parvovirus Thr390Ala Mutant in Egyptian Cats during 2023: Insights from a Comprehensive Full-Length VP2 Genetic Analysis. BMC Vet. Res..

[2] Wang H., Xue L., Wang L., Liu Y., Chen J., Sun Y., An T., Chen H., Yu C., Xia C. (2025). The Quadruplex TaqMan MGB Fluorescent Quantitative PCR Method for Simultaneous Detection of Feline Panleukopenia Virus, Feline Herpesvirus 1, Feline Calicivirus and Feline Infectious Peritonitis Virus. Front. Cell. Infect. Microbiol..

[3] Zhang X., Zhao H., Lu H., Wang R., Liu H., Wang Y., Xing M. (2025). Isolation and Characterization of a Novel Feline Panleukopenia Virus Strain from Amur Tigers: Insights into Pathogenesis and Diagnostic Development. Res. Vet. Sci..

[4] Feng E., Ye Z., Yan M., Zhou Y., Wu D., Cheng S., Cheng Y. (2025). Genetic and Biological Properties of an Epidemic Feline Panleukopenia Virus Strain (Ala91Ser) in China. Vet. Sci..

[5] Wang M.Y., Zhao S.B., Wang S.Y., Du M.H., Ming S.L., Zeng L. (2025). Feline Panleukopenia Virus ZZ202303 Strain: Molecular Characterization and Structural Implications of the VP2 Gene Phylogenetic Divergence. Int. J. Mol. Sci..

[6] Li L., Liu Z., Liang R., Yang M., Yan Y., Jiao Y., Jiao Z., Hu X., Li M., Shen Z. (2024). Novel Mutation N588 Residue in the NS1 Protein of Feline Parvovirus Greatly Augments Viral Replication. J. Virol..

[7] Zhang H., Zhang W., Pan Y., Li H., He T., Dong Q., Song W., Zhang W., Zhang L., Kareem K. (2024). Evolutionary Dynamics and Pathogenicity Analysis of Feline Panleukopenia Virus in Xinjiang, China. Microorganisms.

[8] Franzo G., Tucciarone C.M., Cecchinato M., Drigo M. (2017). Canine Parvovirus Type 2 (CPV-2) and Feline Panleukopenia Virus (FPV) Codon Bias Analysis Reveals a Progressive Adaptation to the New Niche after the Host Jump. Mol. Phylogenet. Evol..

[9] Horiuchi M., Yamaguchi Y., Gojobori T., Mochizuki M., Nagasawa H., Toyoda Y., Ishiguro N., Shinagawa M. (1998). Differences in the Evolutionary Pattern of Feline Panleukopenia Virus and Canine Parvovirus. Virology.

[10] Chen X., Wang J., Zhou Y., Yue H., Zhou N., Tang C. (2022). Circulation of Heterogeneous Carnivore Protoparvovirus 1 in Diarrheal Cats and Prevalence of an A91S Feline Panleukopenia Virus Variant in China. Transbound. Emerg. Dis..

[11] Hoang M., Wu C.N., Lin C.F., Nguyen H.T.T., Le V.P., Chiou M.T., Lin C.N. (2020). Genetic Characterization of Feline Panleukopenia Virus from Dogs in Vietnam Reveals a Unique Thr101 Mutation in VP2. PeerJ.

[12] Yang S., Wang S., Feng H., Zeng L., Xia Z., Zhang R., Zou X., Wang C., Liu Q., Xia X. (2010). Isolation and Characterization of Feline Panleukopenia Virus from a Diarrheic Monkey. Vet. Microbiol..

[13] Yang Y., Geng Y., Ouyang P., Li Y., Guo H., Deng H., Hou R., Lai W., Zhang D., Liu S. (2023). Identification of a Feline Panleukopenia Virus from Captive Giant Pandas (*Ailuropoda melanoleuca*) and Its Phylogenetic Analysis. Transbound. Emerg. Dis..

[14] Yi S., Liu S., Meng X., Huang P., Cao Z., Jin H., Wang J., Hu G., Lan J., Zhang D. (2021). Feline Panleukopenia Virus with G299E Substitution in the VP2 Protein First Identified from a Captive Giant Panda in China. Front. Cell. Infect. Microbiol..

[15] Fei-Fei D., Yong-Feng Z., Jian-Li W., Xue-Hua W., Kai C., Chuan-Yi L., Shou-Yu G., Jiang S., Zhi-Jing X. (2017). Molecular Characterization of Feline Panleukopenia Virus Isolated from Mink and Its Pathogenesis in Mink. Vet. Microbiol..

[16] Huang S., Li X., Xie W., Guo L., You D., Xu H., Liu D., Wang Y., Hou Z., Zeng X. (2022). Molecular Detection of Parvovirus in Captive Siberian Tigers and Lions in Northeastern China From 2019 to 2021. Front. Microbiol..

[17] Wang K., Du S., Wang Y., Wang S., Luo X., Zhang Y., Liu C., Wang H., Pei Z., Hu G. (2019). Isolation and Identification of Tiger Parvovirus in Captive Siberian Tigers and Phylogenetic Analysis of VP2 Gene. Infect. Genet. Evol..

[18] Wang X., Li T., Liu H., Du J., Zhou F., Dong Y., He X., Li Y., Wang C. (2017). Recombinant Feline Parvovirus Infection of Immunized Tigers in Central China. Emerg. Microbes Infect..

[19] Yeo Y.G., Kim H.R., Park J., Kim J.M., Shin Y.K., Lee K.K., Kwon O.K., Jeoung H.Y., Kang H.E., Ku B.K. (2023). Epidemiological and Molecular Approaches for a Fatal Feline Panleukopenia Virus Infection of Captive Siberian Tigers (*Panthera tigris altaica*) in the Republic of Korea. Animals.

[20] Fang N., Li M., Zhang A., Wen L., Li H., Ye X., Zhang F., Chen R. (2025). Cross-Species Characterization of Feline Parvovirus and Feline-Origin Canine Parvovirus Southern China (2023–2024): Insights into Epidemiology, Genetic Evolution and Recombination. BMC Vet. Res..

[21] Zhao H., Wang J., Jiang Y., Cheng Y., Lin P., Zhu H., Han G., Yi L., Zhang S., Guo L. (2017). Typing of Canine Parvovirus Strains Circulating in North-East China. Transbound. Emerg. Dis..

[22] Cheng N., Zhao Y., Han Q., Zhang W., Xi J., Yu Y., Wang H., Li G., Gao Y., Yang S. (2019). Development of a Reverse Genetics System for a Feline Panleukopenia Virus. Virus Genes.

[23] Zhao S., Hu H., Lan J., Yang Z., Peng Q., Yan L., Luo L., Wu L., Lang Y., Yan Q. (2023). Characterization of a Fatal Feline Panleukopenia Virus Derived from Giant Panda with Broad Cell Tropism and Zoonotic Potential. Front. Immunol..

[24] Inthong N., Kaewmongkol S., Meekhanon N., Sirinarumitr K., Sirinarumitr T. (2020). Dynamic evolution of canine parvovirus in Thailand. Vet. World.

[25] Li J., Peng J., Zeng Y., Wang Y., Li L., Cao Y., Cao L., Chen Q., Ye Z., Zhou D. (2024). Isolation of a Feline-Derived Feline Panleukopenia Virus with an A300P Substitution in the VP2 Protein and Confirmation of Its Pathogenicity in Dogs. Anim. Dis..

[26] Allison A.B., Organtini L.J., Zhang S., Hafenstein S.L., Holmes E.C., Parrish C.R. (2016). Single Mutations in the VP2 300 Loop Region of the Three-Fold Spike of the Carnivore Parvovirus Capsid Can Determine Host Range. J. Virol..

[27] Sehata G., Sato H., Yamanaka M., Takahashi T., Kainuma R., Igarashi T., Oshima S., Noro T., Oishi E. (2017). Substitutions at Residues 300 and 389 of the VP2 Capsid Protein Serve as the Minimal Determinant of Attenuation for Canine Parvovirus Vaccine Strain 9985-46. J. Gen. Virol..

[28] Wang J., Yan Z., Liu H., Wang W., Liu Y., Zhu X., Tian L., Zhao J., Peng Q., Bi Z. (2024). Prevalence and Molecular Evolution of Parvovirus in Cats in Eastern Shandong, China, between 2021 and 2022. Transbound. Emerg. Dis..

[29] Decaro N., Desario C., Miccolupo A., Campolo M., Parisi A., Martella V., Amorisco F., Lucente M.S., Lavazza A., Buonavoglia C. (2008). Genetic Analysis of Feline Panleukopenia Viruses from Cats with Gastroenteritis. J. Gen. Virol..

[30] Demeter Z., Gal J., Palade E.A., Rusvai M. (2009). Feline Parvovirus Infection in an Asian Palm Civet (*Paradoxurus hermaphroditus*). Vet. Rec..

[31] Jeoung S.Y., Ahn S.J., Kim D. (2008). Genetic Analysis of Feline Panleukopenia Virus (FPLV) from Cats in Korea. Cornell Hosp. Q..

[32] BukarKolo Y., Buba E., Igbokwe I., Egwu G. (2018). Prevalence of Feline Panleukopenia Virus in Pet and Stray Cats and Associated Risk Factors in Maiduguri, Nigeria. Alex. J. Vet. Sci..

[33] Dang T.T.M., Tran T.T., Van T.M., Le Q.T., Tran Ngoc B. (2023). First Molecular Report of Feline Panleukopenia Virus Infection in Diarrheic Cats at Can Tho City, Vietnam. Vet. Integr. Sci..

[34] Tucciarone C.M., Franzo G., Legnardi M., Lazzaro E., Zoia A., Petini M., Furlanello T., Caldin M., Cecchinato M., Drigo M. (2021). Genetic Insights into Feline Parvovirus: Evaluation of Viral Evolutionary Patterns and Association between Phylogeny and Clinical Variables. Viruses.

[35] Su X., Zhou H., Jiang W., Xu F., Xiao B., Zhang J., Qi Q., Yang B. (2026). Isolation and Evolutionary Analysis of Feline Panleukopenia Virus Strains FPV-BJ-J2 and FPV-BJ-J3 (T440A, N564S, A568G) in Beijing, China. Front. Vet. Sci..

[36] Xie Q., Sun Z., Xue X., Pan Y., Zhen S., Liu Y., Zhan J., Jiang L., Zhang J., Zhu H. (2024). China-Origin G1 Group Isolate FPV072 Exhibits Higher Infectivity and Pathogenicity than G2 Group Isolate FPV027. Front. Vet. Sci..

[37] Pan S., Man Y., Xu X., Ji J., Zhang S., Huang H., Li Y., Bi Y., Yao L. (2024). Genetic Diversity and Recombination Analysis of Canine Parvoviruses Prevalent in Central and Eastern China, from 2020 to 2023. Microorganisms.

